# Regulation of R-loops by nucleic acid and protein modifications

**DOI:** 10.1042/EBC20253035

**Published:** 2025-09-04

**Authors:** Yiyun Zhang, Xiaoyun Zheng, Sumin Ye, Yijia Ma, Jianping Jin

**Affiliations:** 1 Life Sciences Institute, Zhejiang University, Hangzhou, Zhejiang Province 310058, China; 2 Center for Life Sciences, Shaoxing Institute, Zhejiang University, Shaoxing, Zhejiang Province 321000, China

**Keywords:** DNA damage, genome integrity, post translation modification, R-loop, ubiquitins

## Abstract

R-loop, a three-stranded nucleic acid structure consisting of the RNA:DNA hybrid and the displaced singlestranded DNA, is crucial for many cellular processes but could be a threat to genome integrity if dysregulated. The homeostasis of R-loops is governed by various factors including helicases, nucleases, and chromatin remodelers. Since there are many excellent reviews about R-loops, we focus on discussing how R-loop homeostasis is regulated via nucleic acid and protein modifications. We summarize how RNA modifications such as N6-methyladenosine (m^6^A), N5-methylcytosine (m^5^C), and potentially 3-methylcytidine (m^3^C), alongside DNA modifications such as deamination, methylation, and oxidation, influence R-loop dynamics. Moreover, we discuss how protein modifications, including ubiquitination, SUMOylation, acetylation, methylation, and phosphorylation, modulate the activity, stability, or recruitment of R-loop processing factors. Importantly, these modifications often interact with each other and exhibit context-dependent roles, either promoting R-loop formation or facilitating resolution. Elucidating how these chemical codes orchestrate R-loop homeostasis will facilitate our understanding of the mechanisms governing R-loop homeostasis and could provide some insights into genome maintenance, gene expression, and pathogenesis caused by R-loop dysregulation.

## Introduction

R-loops are three-stranded nucleic acid structures consisting of an RNA:DNA hybrid and the displaced single-stranded DNA [1]. While physiological R-loops are important for cellular activities such as transcription regulation [2,3], immunoglobulin class switching [4], and telomere maintenance [5,6], aberrant R-loop accumulation can cause replication stress, DNA damage, and genome instability, potentially leading to various diseases including cancers and neurodegenerative disorders [7]. These dynamic structures emerge through multiple routes, including RNA polymerase pausing during transcription elongation, sequence-specific R-loop initiation motifs (e.g., GC-rich regions), or chromatin context-dependent hybridization events [8]. While evolutionary conservation across species suggests functional importance, their persistence requires strict spatiotemporal control. Cells have evolved numerous regulatory mechanisms involving factors such as helicases, nucleases, topoisomerases, and chromatin remodelers to tightly control R-loop homeostasis [9–12]. For example, THO is a multi-subunit protein complex that facilitates coupling between transcription and mRNA processing. It mediates mRNP packaging and then prevents RNA:DNA hybrid formation [13]. Resolvers such as endoribonuclease RNase H enzymes specifically degrade RNA:DNA hybrids [9], while helicases like Senataxin (SETX) unwind R-loops through ATP-dependent 5′→3′ translocation [12]. DNA topoisomerase like Top1 regulates R-loop formation owing to its ability to resolve both positive and negative supercoils [11]. Besides protein factors, modifications to the displaced single-stranded DNA or the RNA strand of R-loops can either promote R-loop formation or recruit its degradation factors [14–16]. R-loops inevitably induce DNA damage responses. Thus, R-loop clearance is coupled with various DNA repair pathways. For instance, the FA/BRCA pathway repairs R-loop-associated DNA breaks [17]. Recent studies also reveal that chromatin state profoundly influences R-loop dynamics, where H3K36me3-marked active genes exhibit R-loop resistance, while H3K27ac-enriched enhancers show R-loop susceptibility [18]. Therefore, R-loop dynamics is tightly regulated by many factors, including nucleic acid modifications and post-translational modifications (PTMs) of R-loop processing factors.

There are many good reviews about R-loop, its regulation, especially its implications in diseases [19–25]. However, no comprehensive review is available about R-loop regulation by nucleic acid and protein modifications, although nucleic acid modifications were discussed in several recent articles [19–23]. Recent studies have uncovered new regulatory mechanisms of these R-loop processing factors, primarily through post-transcriptional modifications and PTMs [26–28]. Modifications on nucleic acids (both RNA and DNA) and proteins act as critical signaling hubs that fine-tune cellular homeostasis of R-loops [15,29]. These modifications can either alter the properties of R-loops or modulate functions of the proteins which engage in R-loop regulation. Therefore, a systematic review focusing on both nucleic acid modifications and PTMs of R-loop regulatory factors could provide combinational views on implications of the structural components, i.e., DNA and RNA, of R-loops and regulatory factors in maintenance of R-loop homeostasis.

This review summarizes how nucleic acid and protein modifications function as ‘regulators of regulators’ to manage R-loop homeostasis. We will discuss the roles of key RNA modifications (m^6^A, m^5^C, and m^3^C), elucidate how DNA modifications (deamination, methylation, demethylation, oxidative damage) shape R-loop landscapes, and explain how protein modifications (ubiquitination, SUMOylation, acetylation, methylation) orchestrate the activity of R-loop processing factors. These high-degree signaling networks highlight the complexity of R-loop regulation, often in a context-dependent manner, underscoring its importance for genomic integrity maintenance and other cellular functions. Importantly, this review emphasizes the interplay among the nucleic acid modifications and various PTMs, potentially leading to a new research angle for R-loop regulations.

## Regulation of R-loops by DNA modifications

DNA modifications refer to the reversible chemical modifications of DNA bases or accessible sites without altering the DNA sequence. These modifications regulate gene expression, cell fate, and disease occurrence by influencing DNA structure or protein binding [30]. At least 17 chemical modifications have been discovered in DNA, which play significant roles in various biological processes and diseases, including development, aging, and cancers [31]. These modifications do not interfere with Watson–Crick pairing, but they do affect DNA–protein interactions in the double helix groove [31]. Thus far, only a few DNA modifications, such as methylation, deamination, and 8-oxoG modification, have been implicated in R-loop regulation. We will elaborate on their roles in R-loop regulation in detail and discuss the related molecular mechanisms.

### Regulation of R-loops by DNA methylation

**Figure 1:**
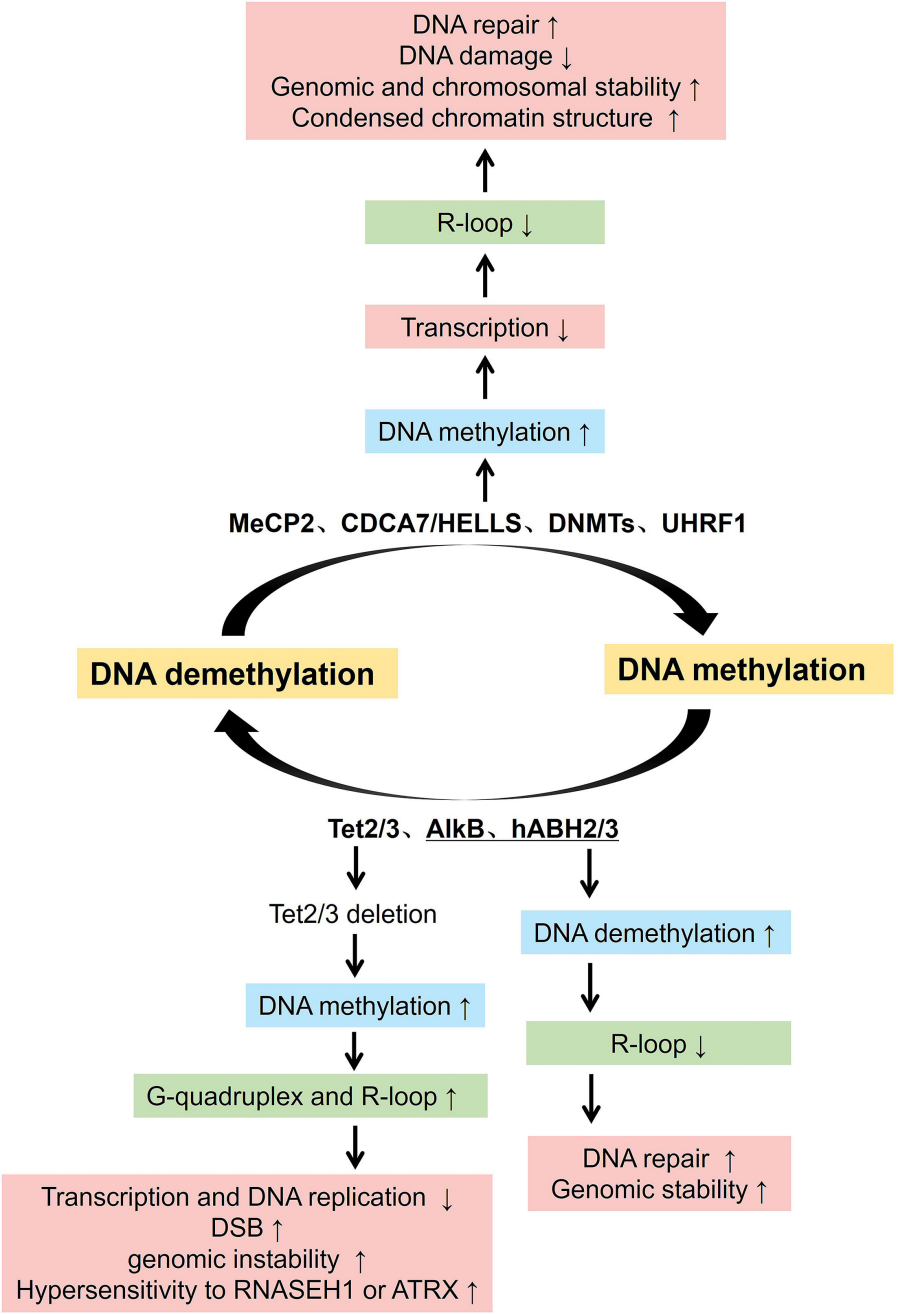
Regulation of R-loops by DNA methylation and demethylation. R-loop is regulated by the intricate balance between DNA methylation and demethylation. Methylation is mediated by MeCP2, CDCA7, HELLS, DNMTs, and UHRF1, and so on. Increased DNA methylation leads to a decrease in transcription, which in turn affects the R-loop formation. The reduction in R-loops promotes DNA repair, decreases DNA damage, enhances genomic and chromosomal stability, and results in a condensed chromatin structure. Demethylation is mediated by the TET family, AlkB family, and hABH2/3. They generally resolve R-loop-associated methylation damage, thus leading to DNA repair and genomic stability. The deletion of TET2/3 increases DNA methylation, leading to the formation of G-quadruplexes and R-loops. Tet-deficient B cells show increased hypersensitivity to RNase H1 and ATRX, suggesting potential vulnerabilities in the DNA repair mechanism. Unlike TET, increased DNA demethylation by AlkB family and hABH2/3 results in decreased R-loop formation, which enhances DNA repair and genomic stability.

In the mammalian genome, methylation of the fifth carbon of cytosine (5-methylcytosine, abbreviated as 5mC) is the most significant DNA modification and is an epigenetic marker associated with gene silencing [32]. DNA methylation, particularly at CpG islands and repetitive sequences, generally correlates with compact chromatin and transcriptional repression, thereby limiting R-loop formation (Figure 1). Hypomethylation of pericentromeric repeats allows aberrant transcription and R-loop accumulation, driving genomic instability. Several factors link DNA methylation to R-loop suppression. For instance, the CDCA7/HELLS chromatin remodeling complex recruits DNA methyltransferases DNMT1 and chromatin modifier UHRF1 (ubiquitin like with plant homeodomain and ring finger domains 1) during replication to maintain DNA methylation patterns, preventing R-loop formation and associated DNA double-strand breaks (DSBs) at heterochromatic regions [33–36]. Defects in this complex cause an immunodeficiency, centromeric instability, and facial anomalies (ICF) syndrome, underscoring its importance [33]. Similarly, MeCP2, a methyl-CpG-binding protein mutated in Rett syndrome, compacts chromatin at methylated sites, restricting R-loop formation [37]. The *de novo* methyltransferase DNMT3b targets centromeric GC-rich regions and restricts R-loop accumulation at pericentromeric repeats [38,39]. Therefore, it is not surprising to see DNMT3b dysfunction leading to R-loop-driven centromeric fragility, which is mediated by XPG/XPF endonucleases, thereby promoting chromosomal instability [40–42]. However, whether DNMT3b loss directly increases R-loop formation or primarily sensitizes existing hybrids for cleavage needs further clarification.

In plants, analogous mechanisms exist. The UHRF1 homolog VIM1 (Variant in Methylation 1), a member of a small gene family which encodes proteins containing PHD, RING, and SRA (SET- and RING-associated) domains, maintains DNA methylation [43]. The lncRNA *APOLO* (Auxin-Regulated Promoter Loop) forms R-loops at the *YUCCA2* promoter, recruiting VIM1 and the polycomb repressive complex 1 component LHP1 to mediate DNA methylation and H3K27me3 deposition, thereby repressing auxin biosynthesis [44]. The fact that the human lncRNA *UPAT* (UHRF1 Protein Associated Transcript) interacts with VIM1/LHP1 in plant cells suggests evolutionary conservation of lncRNA-scaffolded, R-loop-mediated epigenetic regulation, likely dependent on structure rather than sequence [44].

### Regulation of R-loops by DNA demethylation

Active DNA demethylation, primarily mediated by the TET family enzymes that oxidize 5-methylcytosine (5mC), also dynamically regulates R-loop homeostasis (Figure 1) [45–47]. TET enzymes (TET1/2/3) convert 5mC through successive oxidation steps (5hmC→5fC→5caC), facilitating demethylation and maintaining dynamic epigenetic landscapes [47–52]. In B cells, TET2/3 deletion induces hypermethylation, promoting G-quadruplex and R-loop accumulation at immunoglobulin switch regions [52,53]. These non-B DNA structures stall replication forks and recruit AID (activation-induced cytidine deaminase), exacerbating DSBs and contributing to lymphomagenesis [51,54–58]. Tet-deficient B cells show increased sensitivity to depletion of R-loop resolving factors such as RNase H1 and ATRX, suggesting these proteins are potential therapeutic targets [53]. The interplay between methylation (DNMT1) and demethylation (TETs) is highlighted by the rescue of R-loop accumulation in Tet-null models upon DNMT1 deletion. However, whether TET deficiency promotes R-loops directly via chromatin relaxation or indirectly through transcription–replication-induced R-loop formation needs further study. The bacterial AlkB family demethylases and their mammalian homologs (hABH2/3) target RNA:DNA hybrids as well, resolving R-loop-associated methylation damage, indicating conserved roles of demethylases in R-loop resolution [59–64].

Conversely, R-loops can act as platforms to recruit demethylation machinery. The RNA helicase DHX33 can interact with the transcription factor AP-2β and GADD45A (growth arrest and DNA damage protein 45A) to target CpG island promoters via R-loops, recruiting TET1 for demethylation [65]. Genome-wide studies have confirmed thousands of R-loop-dependent TET1-binding sites in embryonic stem cells, implicating that R-loops direct demethylation to specific genomic loci [65]. DHX33 is a helicase that stabilizes R-loops. Loss of DHX33 disrupts R-loop formation, impairing the efficient recruitment of GADD45A and TET1 to gene promoters. This disruption suppresses DNA demethylation; consequently, it inhibits gene transcription [29,47,65–67]. However, how the structural features of different R-loops might influence the efficiency and specificity of TET recruitment remains an open question.

### Regulation of R-loops by DNA deamination

DNA deamination removes an amine group (-NH_2_) from a nucleotide base within the DNA molecule. The APOBEC3 family of mammalian cytidine deaminases, including APOBEC3G, has been shown to inhibit the synthesis of HBV RNA:DNA hybrids [68]. However, this process is deamination-independent. Intriguingly, adenosine deaminases acting on RNA (ADARs), known for catalyzing adenosine-to-inosine (A-to-I) editing in double-strand RNA (dsRNA), also target the DNA strand of R-loops [69–71]. ADAR1 and ADAR2 can deaminate 2′-deoxyadenosine (dA) to 2′-deoxyinosine (dI) on the DNA strand within the hybrid structure. This dI lesion is recognized and cleaved by endonuclease V, initiating DNA repair or R-loop clearance process [16,71]. ADARs exhibit increased binding affinity for longer hybrids via their dsRNA-binding domains, but their deamination efficiency is higher on A-C mismatched hybrids [71]. This substrate preference positions ADARs as critical guardians against pathogenic R-loop accumulation, implicating them in autoimmune disorders such as Aicardi–Goutières syndrome [72–74].

Notably, the nuclear isoform ADAR1p110 selectively edits telomeric RNA:DNA hybrids, converting unstable A-C mismatches to stable I:C pairs. This process facilitates RNase H2-mediated RNA strand degradation, maintaining telomere stability in cancer cells [16]. ADAR1 deficiency leads to telomeric R-loop accumulation, telomere dysfunction, and genomic instability. In telomerase-dependent cancers, ADAR1p110 suppresses telomeric R-loops to support unlimited proliferation, revealing its context-dependent oncogenic role [16]. Importantly, ADAR1 exists in two isoforms: the cytoplasmic form (p150) regulates immune evasion, while the nuclear one (p110) safeguards telomere stability through hybrid editing [16]. This duality suggests ADAR1 inhibitors could exert dual anti-tumor effects—disrupting both immune evasion (via p150) and telomere maintenance (via p110). Therefore, therapeutic targeting of ADARs requires cautious balance, hoping to suppress tumor growth while still preserving R-loop homeostasis in normal cells. In the future, new strategies are needed to employ either isoform-specific inhibitors or RNase H activators to enhance treatment specificity.

### Regulation of R-loops by 8-oxoG modifications

Oxidative stress generates mutagenic lesions such as 7,8-dihydro-8-oxoguanine (8-oxoG), a hallmark of oxidative DNA damage. While typically removed by the base excision repair (BER) pathway initiated by OGG1 (8-oxoguanine DNA glycosylase-1), 8-oxoG within R-loops presents a unique regulatory mechanism [75–77]. OGG1 is a repair protein for 8-oxoG in eukaryotic atopic DNA, which can bind to 8-oxoG within the DNA strand of an R-loop, but instead of efficiently excising it, this interaction paradoxically stabilizes the R-loop structure, potentially by hindering nascent RNA displacement or repair progression [78]. OGG1 inhibition can alleviate oxidant-driven inflammation, while its activity can exacerbate replication stress in cancer cells containing high levels of oxidative damage, suggesting potential therapeutic strategies targeting BER in specific contexts.

### Integrated roles of DNA modifications

DNA modifications act as both sensors and regulators of R-loops, integrating epigenetic states to balance genome stability and gene expression (Table 1). The context-dependent nature of these modifications highlights an integrated gene regulatory network. In general, methylation suppresses R-loop formation, and demethylation can in turn be guided by R-loops. Damage like 8-oxoG is recognized in ways that stabilize R-loops. Therefore, therapeutic strategies targeting these pathways must consider the multifaceted roles of R-loops and ensure specificity to minimize off-target effects. Future work should focus on dissecting the cross-talk between different DNA modifications and R-loop dynamics in various cellular and disease states.

## Regulation of R-loops by RNA modifications

**Figure 2:**
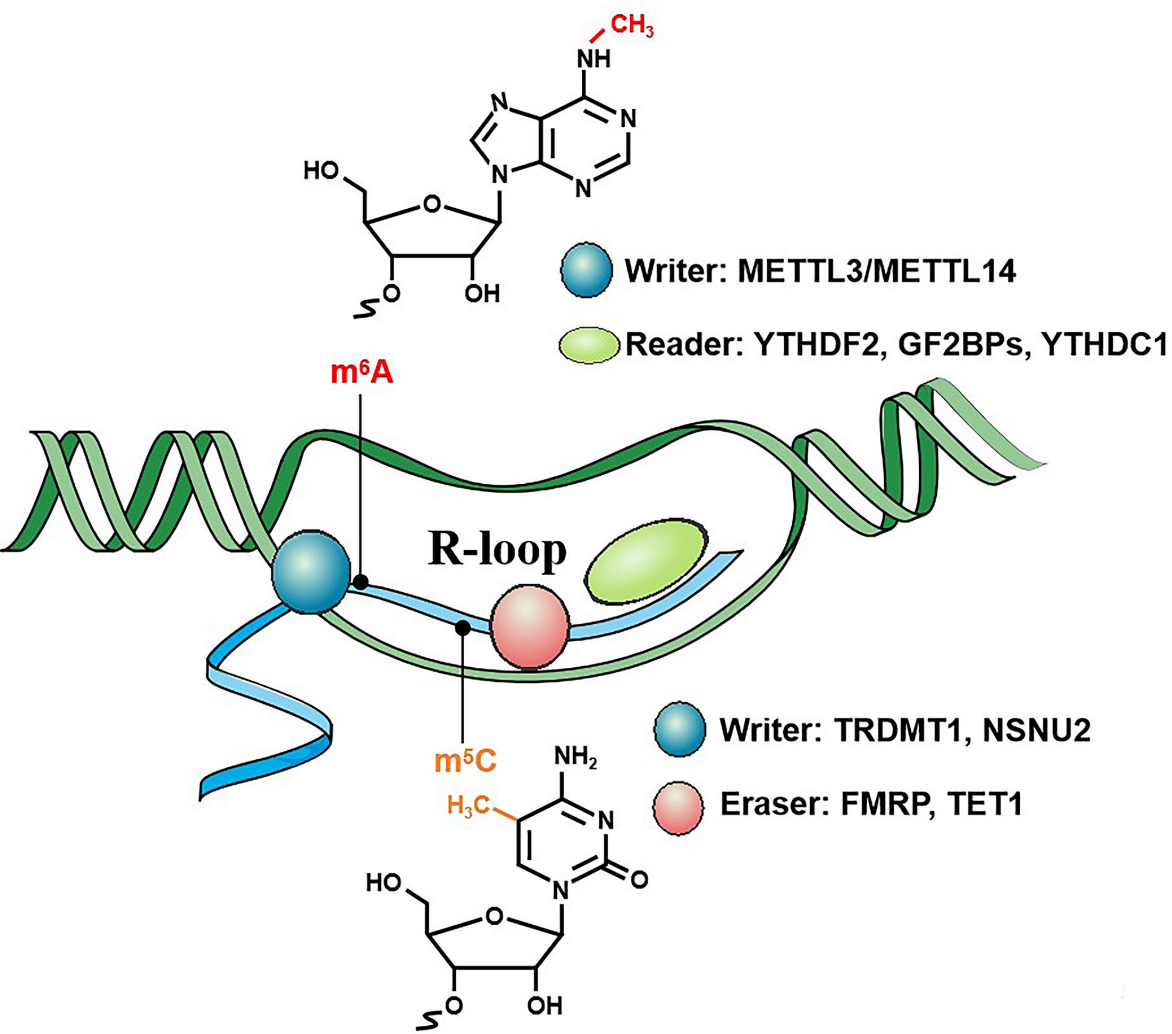
Key RNA methylation writers, erasers, and readers regulating R-loops. The regulation of R-loops by RNA methylation encompasses two distinct post-transcriptional modifications: m^6^A (methylation at the N-6 position of adenosine) and m^5^C (methylation at the fifth carbon of cytosine) and is intricately modulated by key RNA methylation enzymes, including ‘writers’, ‘erasers’, and ‘readers’. The METTL3/14 complex functions as the primary ‘writers’ to catalyze m⁶A modification, while YTHDF2, IGF2BPs, and YTHDC1 serve as distinct ‘readers’ to mediate the biological effects of m⁶A. TRDMT1 and NSUN2 act as ‘writers’ for m⁵C modification, whereas FMRP and TET1 function as ‘erasers’ to dynamically regulate m⁵C levels. These regulators modulate R-loop formation, stability, and resolution through their regulation of RNA modification dynamics, thereby influencing R-loop dynamics and associated biological processes such as transcription, genome stability.

Since the discovery of pseudouridine, a modified nucleoside, over 170 RNA modifications have been identified. These RNA modifications dynamically regulate RNA structure, stability, and interactions with regulatory proteins [80,81]. While initially characterized in tRNA and rRNA, recent studies, particularly on m^6^A in mRNA, have revealed their profound effects on diverse cellular processes, including host–pathogen interactions [82], cell differentiation [83], tumorigenesis [84], and the maintenance of genomic stability [85]. Emerging evidence indicates that RNA modifications, notably m^6^A and m^5^C, are key regulators for RNA:DNA hybrid formation, stability, and resolution, thereby directly affecting R-loop homeostasis (Figure 2) [86].

**Table 1:** Summary of DNA modifications in R-loop regulation

Modification	Regulators	Mechanism	Ref
Deamination	ADAR (ADAR1/2)	ADAR1/2 facilitate the resolution of telomeric R-loops by RNaseH2 to maintain genomic stability and prevent telomeric DNA damage.	[16,71]
Methylation	CDCA7/HELLS	The CDCA7/HELLS complex maintains DNA methylation by recruitment of DNMT1/UHRF1, thereby preventing the accumulation of R-loops.	[34]
	DNMT1, UHRF1	DNMT1 can add methyl groups to the CpG sites and maintain the methylated state of DNA, preventing R-loops formation.	[36,79]
	VIM1	VIM1 maintains DNA methylation and inhibits transcription of target gene, thereby preventing R-loops formation.	[43]
	MeCP2	MeCP2 compacts chromatin at methylated sites, restricting R-loop formation and protecting cells from DNA damage.	[37]
	DNMT3B	DNMT3B prevents the accumulation of R-loops at centromeric and pericentromeric regions, thereby protecting against DNA damage.	[34,39]
Demethylation	TET	TET deletion leads to increased DNA methylation, facilitating accumulation of R-loops and G-quadruplexes, impeding transcription and DNA replication.	[53]
	DHX33	DHX33 recruits GADD45A and TET1 for DNA demethylation via R-loops.	[65]
	GADD45A	GADD45A binds directly to R-loops and then mediates local DNA demethylation by recruiting TET1, thus activating gene expression.	[29]
Oxidative demethylation	AlkB/ALKBH2/ALKBH3	The bacterial AlkB family and their mammalian homologs (hABH2/3) target RNA:DNA hybrids for resolution, resolving R-loop-associated methylation damage.	[59]
8-oxoG	OGG1	OGG1 binds to R-loops under oxidative stress conditions, thereby reducing mRNA levels of TIMP1, leading to an inflammatory response in the lungs.	[78]

### Dual roles of m⁶A methylation in R-loop regulation

m^6^A, the methylation of adenosine at the N-6 position, is a prevalent and reversible RNA modification mediating numerous post-transcriptional RNA processes. Its deposition by methyltransferase ‘writers’ (e.g., METTL3/METTL14 complex) [87,88], removal by demethylase ‘erasers’ (e.g., FTO, ALKBH5) [89,90], and interpretation by ‘reader’ proteins (e.g., YTH domain family, IGF2BPs and HNRNPs) [91–96] collectively regulate RNA splicing, stability, translation, and localization. Initial studies showed colocalization of m^6^A-modified RNAs with R-loops, sensitive to RNase H treatment [15]. Subsequent works confirm m^6^A’s role in regulation of R-loop homeostasis, and its dysregulation leads to aberrant DNA damage responses (DDRs) and various diseases [97–101]. In this section, we systematically examine the context-dependent roles of m^6^A writers, readers, and erasers in R-loop regulation.

### m^6^A writers in R-loop regulation

METTL3, the catalytic core of the primary m^6^A writer complex, exhibits context-dependent roles in R-loop regulation. Following DSBs, ATM-mediated phosphorylation of METTL3 facilitates m^6^A deposition on RNAs, recruiting the reader YTHDC1 to stabilize R-loops at DSB sites, facilitating DNA repair [97]. Similarly, METTL3-mediated m⁶A modification of telomeric repeat-containing RNA (TERRA) promotes R-loop formation and recruits hnRNPA2B1, a m^6^A reader, driving alternative lengthening of telomeres (ALT) in cancers [102,103]. Conversely, METTL3 depletion reduces R-loops near transcription end sites, suggesting m^6^A normally promotes formation of R-loops involving RNA polymerase II pausing or termination [104]. Intriguingly, viral pathogens such as human cytomegalovirus (HCMV) can exploit this by recruiting METTL3 to induce m^6^A-R-loops that suppress specific host gene expression, triggering subsequent pathogenesis [105].

However, m^6^A deposition can also promote R-loop resolution. ARID1A (AT-rich interactive domain 1A) belongs to the SWI/SNF family that regulates gene transcription through chromatin remodeling [106]. ARID1A recruits METTL3-METTL14 complexes to R-loops, where subsequent m^6^A labeling facilitates RNase H1-mediated R-loop resolution, ensuring genome stability during DSB repair [14]. Likewise, TonEBP (tonicity-responsive enhancer binding protein) is a transcriptional regulator and recruits METTL3 to R-loops at DNA break sites, where deposited m^6^A modifications recruit RNase H1 for efficient R-loop clearance [107]. Furthermore, the helicase DDX41 scaffolds METTL14-METTL3-YTHDC1 interactions to promote R-loop removal, a process disrupted in myelodysplastic syndromes due to DDX41 deficiency [101].

The dual role of m^6^A writers in promoting both R-loop stabilization and resolution likely depends on cellular context, protein interactions, and subcellular localization of certain R-loop regulators. However, the precise regulatory mechanisms leading to these opposing outcomes require further investigation. Besides, it is necessary to explore the roles of other m^6^A writers in R-loop regulation.

### m^6^A readers in R-loop regulation

Besides writers, m^6^A reader proteins play crucial roles in interpreting the m^6^A signal on R-loops as well. YTHDF2, a cytoplasmic m⁶A reader, localizes to R-loop-enriched genomic regions during mitosis. Its depletion increases R-loops and DNA damage markers, impairing the cell proliferation [15]. In contrast, the IGF2BP reader family members (IGF2BP1/2/3) stabilize R-loops in an m^6^A-dependent manner. IGF2BPs overexpression enhances R-loop levels and alters DNA methylation, potentially by competing with YTHDF2 for binding to m^6^A-modified R-loops, thus inhibiting R-loop resolution [108]. This highlights a competitive interplay between readers that determines R-loop fates. In glioblastoma stem cells (GSCs), nuclear reader YTHDC1 facilitates the export of m⁶A-modified circular RNA circPOLR2B to the cytoplasm, thus reducing nuclear R-loops formed between circPOLR2B and its parental genomic DNA sequences [109]. This alleviates R-loop-mediated transcriptional repression, promoting POLR2B expression and GSC malignancy. However, this contrasts with YTHDC1’s role in stabilizing R-loops at DNA damage sites, indicating its context-dependent functions, possibly mediated by distinct interaction partners in different cellular compartments or states.

The m^6^A-R-loop axis represents a fine-tuned homeostatic system where writers and readers collectively maintain genomic stability. Dysregulation of this balance is implicated in cancer progression and neurodegeneration. However, how the m^6^A erasers regulate R-loops needs further investigation. Key future directions include identifying specific disease-relevant m^6^A-modified R-loop targets and understanding the precise mechanisms linking m^6^A dysregulation to pathology.

### m^5^C RNA methylation is a key regulator of R-loops in DNA damage response

m^5^C, a conserved RNA modification installed by writers (e.g., NSUN2, TRDMT1), removed by erasers (TET family), and recognized by readers (e.g., ALYREF, RAD52), is emerging as a critical regulator of R-loop homeostasis, particularly during DDR [110]. Upon DSBs, the methyltransferase tRNA-aspartic acid methyltransferase 1 (TRDMT1) is recruited to lesion sites to deposit m^5^C marks on RNA:DNA hybrids, stabilizing R-loops and recruiting homologous recombination (HR) factors such as RAD51/RAD52 [111]. TRDMT1 depletion impairs HR efficiency and increases genomic instability.

However, m^5^C modifications also exhibit context-dependent duality. While TRDMT1-mediated m^5^C stabilizes R-loops for HR, the m^5^C reader, fragile X mental retardation protein (FMRP), a cytoplasmic RNA-binding protein, collaborates with TET enzymes to erase these modifications, promoting R-loop resolution [112]. Loss of FMRP causes m^5^C hypermethylation that induces prolonged R-loop persistence and compromises DSB repair. This antagonistic interplay suggests a spatiotemporal regulatory mechanism where m^5^C ‘writing’ and ‘erasing’ fine-tune R-loop duration to balance repair fidelity. Mechanistically, m^5^C-modified R-loops can suppress alternative non-homologous end joining (alt-NHEJ) by structurally hindering PARP activity, potentially favoring transcription-coupled HR (TC-HR) in active genomic regions. These findings align with recent studies showing NSUN2-mediated m^5^C in rRNA promotes R-loop stabilization at transcription termination sites, preventing replication–transcription conflicts [113]. Further research is needed to understand the tissue-specific roles of different m^5^C writers and the diverse functions of its readers across various cellular contexts.

### m^3^C methylation is a potential R-loop regulator

m^3^C, primarily found at position 32 of cytoplasmic and mitochondrial tRNAs, is crucial for tRNA stability and translation fidelity [114,115]. In humans, distinct methyltransferases catalyze this modification in the cytoplasm (METTL2A/B, METTL6) [116,117] and mitochondria (METTL8) [118]. Emerging evidence, although limited, hints at a much broader regulatory role of m^3^C, potentially extending to R-loop dynamics. For instance, SUMOylated METTL8 can translocate to the nucleolus and surrounding regions, where it stabilizes R-loops using the enzymatic activity of METTL8, contributing to tumorigenesis by altering chromatin organization [119]. In comparison with m^6^A that modifies the RNA strand of R-loop, regulation of R-loops by m^3^C lacks more strong evidence and mechanistic data. The direct relationship between m^3^C deposition and R-loop stabilization, including whether m^3^C modification prevents R-loop recognition by R-loop clearance enzymes or recruits specific readers, remains largely unexplored.

### Roles of RNA modifications and future directions

Both m^6^A and m^5^C modifications play a dual role in the formation and stability of R-loops. These modifications are regulated by ‘writer’, ‘eraser’, and ‘reader’ proteins co-operating with other RNA-binding proteins in certain cases to either stabilize R-loops or reduce their accumulation. For example, METTL3 stabilizes R-loops by adding m^6^A modifications, while it can also collaborate with other proteins such as ARID1A and YTHDC1 to facilitate the resolution of R-loops. This intricate interplay highlights the complexity of RNA modification networks and their significant impacts on R-loop biology. Notably, the dysregulation of R-loop accumulation affects genomic instability and DNA damage repair mechanisms, and so on.The interplay between R-loop dysregulation and abnormal RNA methylation drives genomic instability and transcriptomic dysregulation, intensifying cellular malfunction and promoting pathological development across multiple disease contexts, particularly in cancer progression, neurological degeneration, and hematopoietic malignancies.

While m^6^A and m^5^C are characterized as R-loop regulators, particularly in DDRs (Table 2), the roles of other RNA modifications (e.g., m^3^C, m^7^G, pseudouridine) in R-loop homeostasis remain largely uncharacterized, especially in physiological conditions beyond DNA repair and in various pathological states. Current research often focuses on R-loop stability related to replication stress, leaving its roles in chromatin remodeling, transcription-coupled repair, and broader disease-associated genomic instability less understood. Notably, comprehensive genome-wide studies mapping site-specific RNA modification landscapes in relation to R-loop dynamics across disease models and cell lineages remain scarce. Advanced tools such as the CRISPR-based editing of epitranscriptome combined with R-loop mapping techniques (e.g., DRIP-seq, S9.6 CUT&Tag) can be used to establish links between site-specific RNA modifications and R-loop phenotypes, paving the way for more comprehensive understanding of the mechanisms behind.

## Regulation by protein modifications: ubiquitination and beyond

**Figure 3:**
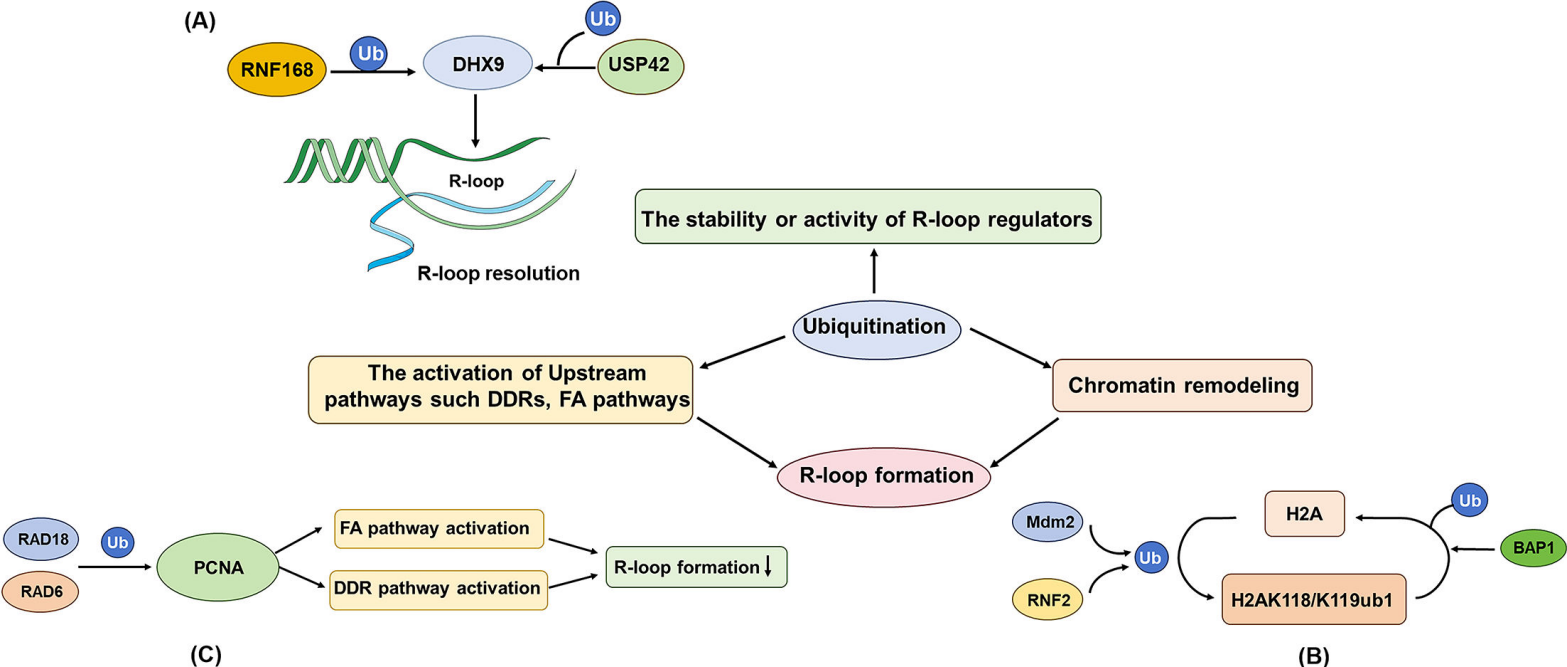
Protein modifications regulate R-loops indirectly. Post-translational modifications such as ubiquitination often affect R-loops through indirect mechanisms. (A) The stability and activity of R-loop regulators are regulated by ubiquitination: for example, RNF168 and USP42 regulate R-loop formation by modulating the ubiquitination of helicase DHX9. (B) R-loop accessibility is regulated by chromatin remodeling due to histone ubiquitination: for example, Mdm2 and RNF2 ubiquitylate histone H2A at residues K118/K119, promoting chromatin condensation and suppressing R-loop formation while BAP1 antagonize this by deubiquitinating H2A. (C) Upstream signaling pathways such as DDRs, FA pathways affect R-loops: for example, RAD18 and RAD6 monoubiquitinates PCNA, facilitating recruitment of DNA repair factors and key FA pathway proteins to prevent R-loop formation and accumulation.

PTMs of proteins add another critical layer of R-loop regulation, directly influencing the functions of helicases, nucleases, chromatin factors, and signaling proteins involved in R-loop metabolism (Figure 3).

**Table 2:** Summary of RNA modifications in R-loop regulation

Modification	Regulator	Partners	Group	Mechanism	Disease/Outcome	Refs
m^6^A	METTL3 / METTL14	ATM / YTHDC1	Writer	ATM phosphorylation of METTL3 facilitates m6A deposition, recruiting YTHDC1 to stabilize R-loops at DSB sites.	DNA repair defects	[97]
		hnRNPA2B1		METTL3-mediated m⁶A modification of TERRA promotes R-loop formation and recruits hnRNPA2B1.	Telomere maintenance and ALT in cancer cells	[102,103]
		-		METTL3 depletion reduces R-loops, disrupts RNA Pol II pausing/termination.	Transcriptional regulation	[104]
		HCMV / UCHL1		Viruses (e.g., HCMV) exploit METTL3 to induce m⁶A-R-loops.	Endothelial injury and viral pathogenesis	[105]
		ARID1A		ARID1A recruits METTL3 complexes to R-loops, labeling R-loops for degradation.	Genome stability	[14]
		TonEBP		TonEBP recruits METTL3 to R-loops at DNA break sites, depositing m6A marks that recruit RNase H1 for R-loop clearance.	Genome stability	[107]
		DDX41		DDX41 deficiency disrupts METTL3-YTHDC1 complex assembly, leading to R-loop accumulation.	MDS	[101]
	YTHDF2	-	Reader	YTHDF2 localizes to R-loop-rich regions during mitosis, preventing replication-associated DNA damage.	Cancer progression defects	[15]
	IGF2BPs	YTHDF2		IGF2BPs compete with YTHDF2 for m⁶A-R-loop binding, thereby preventing R-loop clearance.	Genomic instability and epigenetic dysregulation in cancer	[108]
	YTHDC1	-		YTHDC1 binds m⁶A-modified R-loops at DNA damage sites and stabilizes their structures for repair.	Supports DNA repair	[97]
		*POLR2B*		YTHDC1 promotes cytoplasmic export of m⁶A-modified circPOLR2B in GSCs, reducing nuclear R-loops.	Tumor progression	[109]
m^5^c	TRDMT1	RAD51/RAD52	Writer	TRDMT1 is recruited to DSB sites to deposit m⁵C on R-loops and stabilizes their structures.	Efficient HR repair	[111]
	NSNU2	-		NSNU2 mediates m^5^C in rRNA to resolve R-loops at transcription termination sites.	Bladder cancer progression	[113]
	FMRP / TET1	-	Eraser	FMRP interacts with TET1 to demethylate m⁵C-modified R-loops, thereby facilitating R-loop resolution.	Promotes TC-HR	[112]
m^3^c	METTL8	-	Writer	SUMOylated METTL8 associates with nuclear RNA-binding proteins to promote R-loop formation on ribosomal DNA gene (perhaps through m^3^C).	Tumor progression	[119]

### Introduction of ubiquitin and ubiquitin-like proteins

Ubiquitin (Ub), a 76-amino acid protein is evolutionarily conserved across eukaryotes, and contains seven lysine residues (K6, K11, K27, K29, K33, K48, and K63), which serve as linkage sites for the formation of polyubiquitin chains, creating combinatorial ubiquitin codes with distinct functional outcomes [120]. This reversible PTM is orchestrated by a three-step enzymatic cascade [121]: the ubiquitin-activating enzyme (E1) catalyzes ATP-dependent Ub activation via C-terminal adenylation, followed by transfer of ubiquitin to a ubiquitin-conjugating enzyme (E2), which next co-operates with a ubiquitin ligase (E3) to mediate isopeptide bond formation between ubiquitin and an amino group at the N-terminus of Ub or ε-amino groups of lysine residues on Ub or substrates. Notably, E3 ligases, exceeding 600 in humans, determine substrate specificity through direct recognition of degrons or interaction with adaptor proteins [122]. The tightly regulated ubiquitination process is dynamic and reversible. The dynamic equilibrium of ubiquitination is maintained by over 90 deubiquitinating enzymes (DUBs), which hydrolyze isopeptide bonds to recycle Ub and fine-tune signaling transduction [123].

Ubiquitination regulates diverse cellular processes ranging from proteasomal degradation (primarily via K11/K48-linked chains) to non-proteolytic functions such as DNA repair, kinase activation, and chromatin remodeling, and so on. [124,125]. Recent studies revealed an unexpected role of ubiquitination in regulating R-loop dynamics. For example, proteasome inhibition by bortezomib, which blocks degradation of ubiquitinated substrates, induces R-loop accumulation, suggesting Ub signaling modulates R-loop homeostasis [126]. Ubiquitination affects R-loops primarily by (1) controlling the stability and activity of R-loop regulators (helicases, nucleases, etc.) [26,126,127], (2) modulating chromatin structure affecting R-loop accessibility [128], and (3) regulating upstream signaling pathways such as DDR that influence R-loop levels [129]. Furthermore, ubiquitin-like (Ubl) modifications such as SUMOylation exhibit functional cross-talk with ubiquitination in resolving transcription-associated R-loops [27,130].

### Ubiquitination of R-loop processing factors

Several key R-loop helicases and nucleases are targets of ubiquitination. The E3 ligase RNF168-mediated ubiquitination enhances recruitment of the RNA helicase DHX9 to resolve R-loops [127], while the DUB USP42 removes Ub from DHX9, facilitating its role in BRCA1-dependent R-loop unwinding during HR [131]. The opposing effects of both ubiquitination and deubiquitination on R-loop resolution might reflect regulation by different E3s, Ub chain types, or site-specific modifications directing DHX9 to distinct complexes or functions, highlighting the importance of studying upstream PTMs of R-loop regulatory factors. Similarly, the stability of the helicase SETX is balanced by KEAP1-mediated ubiquitination for degradation and USP11-mediated deubiquitination for stabilization, affecting R-loop levels and associated pathologies [126]. The exonuclease XRN2, crucial for transcription termination and R-loop resolution, is recruited to R-loop sites via RNF8-mediated ubiquitination [26]. Furthermore, the E3 ligase PRP19 co-operates with endonucleases XPF/XPG to recognize and process RNA:DNA hybrids, indicating Ub’s role in both the biogenesis and enzymatic processing of these hybrid structures [132].

### Ubiquitination, chromatin, and R-loops

Chromatin remodeling complexes dynamically regulate R-loop formation and resolution through nucleosome architecture reorganization, transcriptional modulation, and co-ordination with DNA repair machineries. Ubiquitination regulates this by modifying histones and chromatin remodelers. E3 ligases such as Mdm2 and RNF2 ubiquitylate histone H2A at residues K118/K119, which generally promotes chromatin compaction and suppresses R-loop formation, facilitating replication fork progression [128,133]. In contrast, the DUB BAP1 counteracts this by deubiquitinating H2A, creating a switch controlling R-loop accessibility [128]. These findings reveal ubiquitination plays a central role in chromatin-remodeling-mediated R-loop regulation.

### Indirect regulation via DNA damage responses

Ubiquitination regulates R-loop dynamics not only through direct modification of key factors but also by orchestrating signaling cascades that indirectly influence R-loop homeostasis. Pathological R-loop accumulation disrupts DNA replication, repair efficiency, and transcriptional regulation, generating genotoxic stress that in turn reinforces R-loop persistence through unresolved DNA damage [134,135]. The E3 ligase RAD18, with the E2 RAD6, monoubiquitinates PCNA (proliferating cell nuclear antigen), a principal conductor at replication fork co-ordinating multiple DNA repair pathways, at lysine 164. This monoubiquitination event is crucial for recruiting specific DNA repair factors and simultaneously helps prevent R-loop formation during replication stress [129,135]. RAD18-mediated PCNA-Ub facilitates recruitment of FANCD2, a key Fanconi anemia (FA) pathway protein which promotes R-loop resolution through helicase-dependent unwinding [129,136]. On the other hand, the FA pathway’s activation depends on precise monoubiquitination of FANCD2 by the E3 ligase FANCL and the E2 UBE2T, to prevent R-loop accumulation [137], indicating the complexity of Ub signals. Another signaling axis involves the E2 UBE2B and the E3 RAD18 in repairing single-stranded DNA gaps, thereby suppressing R-loop formation, although the exact mechanisms require further elucidation [135].

### Ubiquitin-like modifications (Ubls) in R-loop regulation

Ubls (ubiquitin-like proteins) adapt similar enzymatic cascades to be conjugated on their substrates. Among Ubls, developmentally down-regulated gene 8 (NEDD8), small ubiquitin-like modifier (SUMO), autophagy-related 8 (ATG8), and interferon-stimulated gene 15 (ISG15) are most studied [138,139]. These Ubls orchestrate diverse cellular processes, ranging from DNA repair to transcriptional regulation [125,139]. Intriguingly, emerging evidence positions Ubl-mediated modifications as critical regulators of R-loop homeostasis like Ub.

SUMOylation controls the R-loop resolution by targeting key helicases and topoisomerases. For instance, SUMOylation of DHX9 enhances its interaction with DNA repair-associated protein PARP1 and another helicase DDX21, forming a complex that resolves R-loops and alleviates replication stress [27]. DNA topoisomerase I (TOP1) can also be SUMOylated by the PIAS1–SRSF1 E3 ligase complex at K391/K436 sites, while the helicase RECQ5 interacts with TOP1 and PIAS1–SRSF1 complex through its helicase domain, promoting the SUMOylation of TOP1 [130]. This modification near transcription sites helps suppress R-loop formation during transcription-replication conflicts. While less explored in the R-loop regulation, other Ubls such as NEDD8 and ISG15, known to be involved in replication stress and DNA repair, may also mediate R-loop dynamics by modulating helicase activity or repair factor recruitment.

### Other protein modifications influencing R-loops

Beyond ubiquitination, other PTMs fine-tune R-loop regulation as well. Acetylation is a biochemical process where an acetyl group is added to proteins. It may influence R-loop dynamics by altering chromatin structure or transcriptional activity, thereby affecting genomic stability. In yeast, acetylation augments the helicase activity, ATPase activity, and DNA binding capacities of Pif1, thereby unwinding R-loops [140,141]. Intriguingly, deacetylation of another helicase DDX21 by SIRT7 also augments its helicase activity to unwind R-loops [142], indicating the complexity of acetylation in activity control of helicases involving R-loop regulation. Another example is Histone deacetylase 8 (HDAC8), which counteracts R-loop formation by deacetylating structural maintenance of chromosomes protein 3 (SMC3), thereby preserving chromosomal integrity and replication fork progression [143]. HDAC8 inactivation induces SMC3 hyperacetylation, leading to R-loop accumulation, replication fork stalling, and replication stress. These findings highlight the dual role of acetylation in both promoting and suppressing R-loops, contingent on substrate and cellular context.

R-loops could influence histone modifications by recruiting chromatin-modifying enzymes [144], while specific histone modifications could regulate R-loop formation by altering transcription dynamics and chromatin accessibility [145]. By antagonizing PRC2-mediated H3K27me3 deposition at R-loop regions, ALKBH1 elevates the H3K4me3/H3K27me3 ratio, enabling stress-responsive gene activation and enhancing plant stress tolerance [145]. Another demethylase, PHF2, removes H3K9me2, preventing R-loop-induced DNA damage [146].

Phosphorylation is one of the most studied PTMs where a phosphate group is added to a protein, influencing its structure, functions, or localization. It can regulate the dynamics of R-loops, affecting transcriptional regulation and genomic integrity. For example, BRD4 modulates TOP1 activity by regulating RNAP II CTD phosphorylation, thereby preventing the accumulation of R-loops [147]. Similarly, phosphorylation of RNA helicase DDX23 by SRPK2 is necessary to suppress R-loops [28].

In summary, PTMs represent a critical and complex layer of R-loop regulation (Table 3). We primarily focus on ubiquitination and Ubl modifiers such as SUMOylation but also touch upon acetylation, methylation, and phosphorylation. These PTMs directly modulate the activity and stability of R-loop processing factors, including helicases, nucleases, and topoisomerases. Furthermore, PTMs influence R-loop accessibility and formation by altering chromatin structure, targeting histones, and remodeling complexes. Finally, PTMs, particularly ubiquitination, play essential roles in co-ordinating the DDR signaling cascades that indirectly regulate R-loop resolution and prevention. In conclusion, PTMs are essential for control of R-loop homeostasis and for genomic integrity maintenance via R-loop regulatory factors.

**Table 3:** Summary of protein modifications in R-loop regulation

Modification	Regulators	Mechanism	Ref
Ubiquitination	USP42	USP42 promotes BRCA1-dependent homologous recombination and R-loop unwinding through deubiquitylating DHX9.	[131]
	USP11	USP11 maintains the homeostasis of R-loop by deubiquitylating SETX and antagonizing KEAP1.	[126]
	BAP1	BAP1 deubiquitylates H2A, prevents R-loop formation.	[128]
	RAD6	RAD6 collaborates with RAD18 to monoubiquitinate PCNA and facilitates FANCD2 recruitment, thereby preventing R-loop formation.	[129]
	UBE2T	UBE2T works with FANCL to ubiquitylate FANCD2, thereby preventing R-loop accumulation.	[137]
	RNF168	RNF168 accelerates R-loop resolution by ubiquitinating DHX9 to facilitate its recruitment to R-loop-prone loci.	[127]
	KEAP1	KEAP1 controls R-loops by regulating the stability of R-loop helicase SETX against USP11.	[126]
	RNF8	RNF8 accelerates R-loop resolution by mediating ubiquitination of exonuclease XRN2 to be recruited to R-loop sites.	[26]
	PRP19	PRP19 resolves R-loops by collaborating with endonucleases XPF and XPG.	[132]
	RNF2	RNF2 suppresses R-loop formation by ubiquitylating histone H2A, facilitating DNA replication fork progression.	[128,133]
	MDM2	MDM2 suppresses R-loop formation by ubiquitylating histone H2A, sustaining DNA replication.	[128]
	RAD18	RAD18 collaborates with RAD6 to monoubiquitinate PCNA to facilitate DNA repair and prevent R-loop formation. This also facilitates FANCD2 recruitment, activating FA pathway to promote R-loop resolution.	[129,135,136]
	FANCL	FANCL prevents R-loop accumulation by ubiquitylating FANCD2.	[137]
SUMOylation	PIAS1/SRSF1	PIAS1 works with SRSF1 to mediate TOP1 SUMOylation, thereby inhibiting R-loop formation.	[130]
Acetylation	Pif1	Acetylation of Pif1 promotes the unwinding of R-loops by augmenting its helicase activity.	[140,141]
Deacetylation	SIRT7	Deacetylation of DDX21 by SIRT7 augments its helicase activity to unwind R-loops.	[142]
	HDAC8	HDAC8 deacetylates SMC3 to counteract R-loop formation.	[143]
	ALKBH1	ALKBH1 antagonizes PRC2-mediated H3K27me3 deposition at R-loop regions.	[148]
	PHF2	PHF2 removes H3K9me2 to prevent R-loop-induced DNA damage.	[146]
Phosphorylation	BRD4	BRD4 regulates RNAP II CTD phosphorylation, thereby preventing the accumulation of R-loops.	[149–151]
	SRPK2	SRPK2 phosphorylates DDX23 to suppress R-loop formation.	[28]

## Conclusion and future outlook

In summary, R-loop regulation is a ubiquitous, multi-layered process extending beyond the direct regulation of processing factors. This review underscores the critical roles of chemical modifications on nucleic acids and proteins, which act as ‘regulators of regulators’ to orchestrate R-loop homeostasis. We have elaborated on how RNA modifications (m^6^A, m^5^C, m^3^C), DNA modifications (deamination, methylation, demethylation, oxidation), and protein modifications (ubiquitination, SUMOylation, acetylation, methylation, and phosphorylation) execute complex, often context-dependent control over R-loop formation, stability, and resolution. Interestingly, many modifications could either promote R-loop stabilization or facilitate resolution depending on cellular context, interacting partners, and specific modification sites. This complex network is essential for co-ordinating fundamental processes such as DNA repair, transcription, and replication while safeguarding genomic integrity.

Despite significant progress, critical questions still remain regarding how cross-talk among distinct modifications dynamically orchestrates R-loop homeostasis across physiological or pathological contexts, particularly in light of their dual roles in maintaining genome stability and modulating transcriptional activity. Future research needs advanced genome-wide mapping, such as simultaneous profiling of R-loop and various modifications, and integrated multi-omics approaches. This includes but is not limited to (1) spatiotemporal co-profiling of R-loop dynamics with associated epigenetic modifications (e.g., DNA methylation, histone acetylation, and m^6^A RNA modifications) using simultaneous CUT&Tag/ChIP-seq and DRIP-seq approaches; (2) systematic identification of context-specific writer-reader-eraser circuits through integrated multi-omics analysis combining single-cell epigenomic profiling, CRISPR-based perturbation screens, and proteomic mapping of modification-specific binding partners; (3) functional analysis of cross-talk mechanisms among various modifications using combinatorial knockouts of modifying enzymes coupled with quantitative mass spectrometry analysis of chromatin state transitions. These approaches should be complemented by computational modeling of modification dynamics to identify key points for therapeutic intervention, particularly through pharmacological targeting of pathological R-loop-modification feedback loops. Implementation of such multidimensional strategies will enable mechanistic understanding of how site-specific R-loop-modification interfaces dictate transcriptional outcomes and genome stability in disease states. Systematic analysis will be crucial to elucidate these complex regulatory networks, eventually not only deepening our understanding of fundamental genome biology but also potentially identifying novel therapeutic targets for human diseases driven by R-loop dysregulation.

Thus far, only a few nucleic acid modifications have been linked with regulation of R-loop homeostasis. In fact, at least 17 DNA modifications have been discovered, but only two have been intensively investigated for their roles in R-loop regulation. It will be interesting to see whether other DNA modifications could be involved in control of R-loop homeostasis. A similar situation exists for RNA modifications as well. Equally important, whether other PTMs of R-loop processing factors are important for maintenance of R-loop homeostasis among over 400 different PTMs. Future studies are needed to clarify functions of other modifications of nucleic acids and R-loop regulatory proteins in R-loop control.

**Summary**

- The review emphasizes that R-loop homeostasis is controlled not only by direct regulators (like helicases and nucleases) but also critically by a ‘higher tier’ involving chemical modifications on both nucleic acids (RNA, DNA) and proteins. These modifications act as ‘regulators of regulators’.
- Specific RNA modifications (e.g., m^6^A, m^5^C, m^3^C) and DNA modifications (including deamination, methylation cycling, and 8-oxoG lesions) exert bidirectional regulatory effects on R-loop homeostasis. Notably, these modifications demonstrate context-specific functional dichotomy, either stabilizing R-loop architectures or promoting their dissolution.
- Post-translational modifications (PTMs) such as ubiquitination, SUMOylation, acetylation, phosphorylation, and methylation precisely modulate the activity, stability, or recruitment of the proteins which are directly in processing R-loops.
- The regulatory network governing R-loops via these modifications is complex and highly context-dependent. The specific cellular environment, interacting partners, and locations determine whether a modification promotes or resolves R-loops. The interplays among various modifications could play significant roles in determining the direction of the content-dependent regulation.
- Understanding how these chemical codes orchestrate functions of R-loop regulators is vital for comprehending fundamental processes such as genome maintenance and gene expression, offering critical insights into the pathogenesis of diseases linked to R-loop dysregulation, potentially revealing novel therapeutic targets.

## Open Access

This article has been published open access under our Subscribe to Open programme, made possible through the support of our subscribing institutions, learn more here: https://portlandpress.com/pages/open_access_options_and_prices#conditional

## Abbreviations

**Abbreviations**
ADAR: adenosine deaminases acting on RNA
ALT: alternative lengthening of telomeres
DSB: DNA doublestrand breaks
DUB: deubiquitinating enzyme
E1: ubiquitin-activating enzyme
E2: ubiquitin-conjugating enzyme
E3: ubiquitin ligase
FMRP: Fragile X mental retardation protein
GSC: glioblastoma stem cells
HCMV: human cytomegalovirus
HR: homologous recombination
NEDD8: developmentally down-regulated gene 8
OGG1: 8-oxoguanine DNA glycosylase-1
PTM: Post-translational modification
TERRA: telomeric repeatcontaining RNA
TRDMT1: tRNA-aspartic acid methyltransferase 1
TonEBP: tonicity-responsive enhancer binding protein
Ub: ubiquitin
Ubl: ubiquitin-like protein
dsRNA: double strand RNA
m6A: N6-methyladenosine
m3C: 3-methylcytidine
5mC: 5-methylcytosine
m5C: N5-methylcytosine
8-oxoG: 7,8-dihydro-8-oxoguanine
